# Oxytocinergic Signaling in Zebrafish: Translational Perspectives for Autism Spectrum Disorder

**DOI:** 10.1111/jnc.70346

**Published:** 2026-01-13

**Authors:** Géssica Peres, Melissa Talita Wiprich, Darlan Gusso, Carla Denise Bonan

**Affiliations:** ^1^ Programa de Pós‐Graduação em Medicina e Ciências da Saúde, Escola de Medicina Pontifícia Universidade Católica do Rio Grande do Sul Porto Alegre RS Brazil; ^2^ Programa de Pós‐Graduação em Ciências da Saúde Universidade do Extremo Sul Catarinense (UNESC) Criciúma SC Brazil; ^3^ Programa de Pós‐Graduação em Biologia Celular e Molecular, Escola de Ciências da Saúde e da Vida Pontifícia Universidade Católica do Rio Grande do Sul Porto Alegre RS Brazil

**Keywords:** autism, neurodevelopmental disorders, oxytocin, social behavior, zebrafish

## Abstract

Alterations in the oxytocin system, accompanied by cognitive and behavioral deficits, are common in several neurodevelopmental conditions, including Autism Spectrum Disorder. Oxytocin, a neuropeptide produced in the hypothalamus, plays a pivotal role in modulating social cognition and complex social behaviors. Recently, increasing attention has been given to the therapeutic potential of oxytocin in the treatment of neurodevelopmental disorders. However, many aspects of oxytocin signaling and its effects remain to be fully elucidated. Given its pronounced social behaviors and conserved neurochemical pathways, the zebrafish ( *Danio rerio* ) has emerged as a model for investigating the neural and behavioral effects of oxytocin. This species exhibits a wide behavioral repertoire, making it suitable for modeling oxytocin‐related neurodevelopmental alterations. Here we provide an overview of the key mechanisms underlying oxytocin signaling and discuss current findings supporting the use of zebrafish as an Autism Spectrum Disorder model.

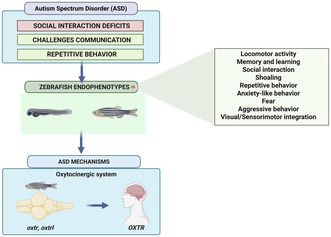

## Graphical abstract

Alterations in oxytocin signaling are strongly implicated in the social and cognitive deficits characterizing Autism Spectrum Disorder (ASD) and other neurodevelopmental conditions. However, the precise mechanisms remain unclear. The zebrafish ( *Danio rerio* ) has emerged as a powerful translational model due to its evolutionarily conserved oxytocin pathways and rich repertoire of complex social behaviors. This review synthesizes key mechanisms of oxytocin signaling and evaluates the evidence supporting the use of zebrafish to model ASD and elucidate these critical pathways.

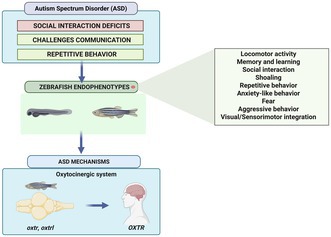

**Abbreviations**
3R's: replacement, reduction and refinement
adsl: adenylosuccinate lyase
aNT: anterior tuberal nucleus
ASD: Autism Spectrum Disorder
CC: crista cerebralis
CCe: cerebellar body
CNS: central nervous system
CNTNAP2: contactin‐associated Protein‐like 2
D: dorsal telencephalic area
DIL: diffuse nucleus of the inferior lobe
Dl: lateral pallium
Dm: medial pallium
dpf: days post fertilization
DSM: Diagnostic and Statistical Manual of Mental Disorders
DSM‐5: Diagnostic and Statistical Manual of Mental Disorders, Fifth Edition
E2: estradiol
ER: estrogen receptor
H: hypothalamus
hpf: hours post‐fertilization
ICD‐11: International Classification of Diseases, Eleventh Revision
LC: locus coeruleus
mbd5a: methyl‐CpG‐binding domain protein 5a
MO: medulla oblongata
MS: medial septum
NDDs: neurodevelopmental disorders
NOR: novel object recognition
NTT: novel tank test
OB: olfactory bulb
OMR: optomotor response
ON: optic nerve
OXTR: oxytocin receptor
OXTRL: oxytocin receptor‐like
OXTRs: oxytocin receptors
PAG: periaqueductal gray substance
POA: preoptic area
R: raphe nucleus
SC: spinal cord
SDMN: social decision‐making network
SHANK3: multiple ankyrin repeat domains 3
Tel: telencephalon
TeO: optic tectum
TPp: periventricular nucleus of the posterior tuberculum
tsb1b: tubulin beta 1b
V: ventral telencephalic area
Vc: central part of subpallium
Vd: dorsal nucleus of subpallium
Vl: lateral subpallium
VnT: ventral tuberal nucleus
VPA: sodium valproate
Vs: supracommissural nucleus of subpallium
VT: ventral thalamus
Vv: ventral part of subpallium
wpf: weeks post‐fertilization
WT: wild‐type
μM: micromolar

## 1 Introduction

Social behavior is essential for the survival and organization of many species, as it depends on dynamic interactions between individuals (Snyder‐Mackler et al. 2020). Oxytocin, a neuropeptide first described by Sir Henry Dale over a century ago, plays a key role in modulating these behaviors (Dale 1906, 1909). This multifunctional molecule has attracted growing interest from various scientific fields, particularly social neuroscience. Since the 1980s, pioneering studies have demonstrated the influence of oxytocin on social behavior, establishing it as a key target in the study of social cognition and neurodevelopmental conditions (Kovács et al. 1978; Sarnyai et al. 1991; Erdozain and Peñagarikano 2020). In this context, oxytocin acts as an important modulator from the central nervous system (CNS), playing an essential role in a wide range of social functions, including: (i) maternal, affiliative, and sexual behavior; (ii) processing of social, emotional, and sensory information; (iii) social cognition and recognition; (iv) attenuation of anxiety; and (v) regulation of fear and stress responses (Choe et al. 2015; De Dreu and Kret 2016; Raam et al. 2017; Menon et al. 2018; Rogers‐Carter et al. 2018; Hirota et al. 2020).

A clinical study in healthy men (aged 20–30 years) demonstrated that the oxytocin system modulates many aspects of social behavior; among these, it increases empathy and promotes feelings of self‐confidence (Colonnello and Heinrichs 2014). In adult men, oxytocin also promotes the conformity of opinions among the members of a social group and plays a fundamental role in the development of affective social bonds (Huang et al. 2015). Evidence from randomized clinical trials in humans and review studies indicates that this neuropeptide also participates in the regulation of social stress and is associated with mentalization, empathy, and altruism processes (Hurlemann et al. 2010; Pohl et al. 2019; Xu et al. 2019). Corroborating these findings, in healthy humans, intranasal administration of oxytocin has positive effects on the perception and emotional recognition of facial expressions, improves eye contact, and modulates cooperation and defense behaviors towards in‐group or out‐group members (Huang et al. 2015; Tillman et al. 2019). In the context of intergroup relations, there is evidence that oxytocin effects depend on the social context and on individual characteristics, such as personality traits, gender, and psychopathological conditions (Shamay‐Tsoory and Abu‐Akel 2016).

In mammals, oxytocin is synthesized in the paraventricular and supraoptic nuclei of the hypothalamus. It is then released via axons to the neurohypophysis, where it is stored and then secreted into the bloodstream to exert physiological functions related to the endocrine system (Neumann 2007; Pohl et al. 2019; Perisic et al. 2024). Oxytocin mediates its effects by binding to oxytocin receptors (OXTRs), a class of metabotropic G protein‐coupled receptors (Song and Albers 2018) expressed in both CNS and peripheral tissues (Jurek and Neumann 2018; Newmaster et al. 2020). In the peripheral system, OXTRs are expressed in organs such as the uterus, kidneys, thymus, bones, and heart, where they regulate several physiological functions, including circadian rhythm, heart rate, osteogenesis, myogenesis, and the modulation of the immune and reproductive functions (Jurek and Neumann 2018; Newmaster et al. 2020). In the CNS, OXTRs are expressed in neurons and glial cells of the hippocampus, amygdala, and prefrontal cortex (Quintana et al. 2019). Evidence indicates that oxytocin signaling begins early in development, particularly during the neonatal period, playing a critical role in brain organization (Muscatelli et al. 2022). Corroborating this, peaks in OXTR expression in cortical areas coincide with critical periods of postnatal development and are essential for lifelong social learning (Vaidyanathan and Hammock 2017). In the hippocampus and amygdala, OXTRs play a key role in regulating social recognition, social learning, and emotional functions. This occurs because the distribution of OXTRs in the ventral and dorsal hippocampus regulates emotional and cognitive processes, respectively, with the ventral region associated with emotion and the dorsal region with social and spatial learning (Walia et al. 2024). The amygdala also contributes to social regulation and is a critical structure for emotional processing, where OXTR activation helps modulate aggressive behavior (Gulevich et al. 2019). Impairments in social recognition and increased aggression—both linked to alterations in oxytocin signaling—are commonly observed in individuals with Autism Spectrum Disorder (ASD) (Zhan et al. 2024; Patwardhan and Choe 2025).

ASD is a multifaceted neurodevelopmental disorder marked by challenges in communication and social interaction. Since its first descriptions (Kanner 1943; Asperger 1944), the concept of autism has expanded considerably. Successive editions of the Diagnostic and Statistical Manual of Mental Disorders (DSM) have refined its definition, culminating in the DSM‐5, which unified previous subtypes under ASD, recognizing a broad spectrum of behavioral, cognitive, and social manifestations (American Psychiatric Association 2013). A systematic review reported an ASD prevalence of 100 per 10 000 individuals (range: 1.09–436.0/10000). The median male‐to‐female ratio was 4.2, and approximately 33% of ASD cases presented co‐occurring intellectual disability (Zeidan et al. 2022). The observed increase in prevalence over time likely reflects the combined effects of increased awareness, improved diagnostic criteria, public health strategies, and enhanced community capacity, rather than solely biological determinants. While epidemiological studies indicate a growing global prevalence of ASD, genetic factors also play a crucial role in its etiology.

Among the several genes implicated in ASD, some are involved in synapse formation, such as *SHANK3* (multiple ankyrin repeat domains 3) (Durand et al. 2007) and *CNTNAP2* (contactin‐associated protein‐like 2) (Zweier et al. 2009). *CNTNAP2* encodes a protein essential for neuronal synapse formation and communication between neurons and has been associated with ASD. Disruption of these genes may impair synaptic connectivity in brain regions critical for social cognition (Kareklas, Teles, Dreosti et al. 2023; Liu et al. 2018). In parallel, oxytocin, a neuropeptide widely recognized for its role in modulating social bonding, affiliative behavior, and conspecific recognition, acts upon these same neural pathways (Zelmanoff et al. 2025). Emerging evidence suggests that alterations in synaptic genes may converge with dysregulated oxytocin signaling, offering a potential mechanistic link between genetic vulnerability and social behavior deficits in ASD (Pekarek et al. 2022; Rokicki et al. 2022).

In fact, it is well established in literature that disturbances in the oxytocinergic system are intimately connected to several psychiatric diseases and neurodevelopmental disorders, including schizophrenia, psychopathy, Prader‐Willi syndrome, and ASD (Grinevich et al. 2015; Rajamani et al. 2018; Peled‐Avron et al. 2020; Rijnders et al. 2021; Goh and Lu 2022). In this context, the zebrafish ( *Danio rerio* ) has emerged as a powerful model organism in diverse research fields, especially in behavioral neuroscience and neuropharmacology for elucidating molecular mechanisms underlying diseases (Qin et al. 2014; Bao et al. 2019). This species displays transparent embryos during early developmental stages, has a fully sequenced genome and shares high physiological, neuroanatomical, and genetic homology with humans (Panula et al. 2010). In addition, this teleost has well‐characterized neurotransmitter systems, including dopaminergic, serotoninergic, purinergic, noradrenaline, oxytocinergic, and endocannabinoid pathways, among others (Rico et al. 2011; Ruhl et al. 2017; Shams et al. 2017; Nabinger et al. 2020; Nunes et al. 2021). Zebrafish are highly social animals capable of distinguishing between familiar and unfamiliar conspecifics (Madeira and Oliveira 2017). They exhibit shoaling preferences as early as 7 days post‐fertilization (dpf), with this behavior becoming increasingly pronounced during development (Hinz and de Polavieja 2017). Due to their social behavior, zebrafish is an excellent organism model for investigating neural mechanisms underlying social behavior and the pathophysiology of neurodevelopmental disorders, such as ASD (Kalueff et al. 2014; Tunbak et al. 2020). Studies using zebrafish and rodents as animal models have shown that oxytocin boosts social learning, induces consolation behavior, increases preferences for conspecifics, regulates recognition of new events, and is implicated in the emotional contagion of fear and in reduction of stress and anxiety responses during social interactions (Burkett et al. 2016; Johnson et al. 2017; Li et al. 2019; Landin et al. 2020; Ribeiro, Nunes, Gliksberg, et al. 2020; Akinrinade, Kareklas, et al. 2023).

Although there is a growing interest in the oxytocinergic system due to its essential role in modulating social behavior, comprehensive reviews focusing on this system in zebrafish, particularly its influence on social behavior in ASD zebrafish models, remain limited. In this review, we discuss the oxytocin‐mediated modulation of social behavior in zebrafish, and the interplay between the oxytocinergic system and ASD models in this species.

## 2 Oxytocin System in Zebrafish

Phylogenetic analyses have suggested that neuropeptides evolved approximately six hundred million years ago through gene duplication events from the ancestral molecule arginine‐vasotocin (Acher and Chauvet 1995). This duplication event created two distinct genes: one produces vasopressin peptides and the other oxytocin (Goodson 2013; Wircer et al. 2016). In zebrafish, isotocin (the homolog of oxytocin) and vasotocin (the homolog of vasopressin) are synthesized by magnocellular and parvocellular neurons located in the preoptic area. Additionally, the magnocellular neurons project to the pituitary to release these neurohormones, which have a key role in social behavior (Grinevich et al. 2016; Herget et al. 2014; Knobloch and Grinevich 2014; Langova et al. 2020).

Due to the genetic duplication process, zebrafish have two types of orthologous oxytocin receptors (OXTRs): oxytocin receptors (OXTR) and oxytocin receptor‐like (OXTRL) (Nunes et al. 2020). These receptors are both biologically active and play essential roles in regulating social behavior (Landin et al. 2020). Although they are paralogs rather than strict orthologs of the mammalian OXTR, studies indicate that they do not act redundantly. Landin et al. (2020) demonstrated that both receptors are pharmacologically targeted by the non‐peptidergic antagonist L‐368899, reinforcing their functional conservation. Knockout studies have shown that the absence of either receptor affects social behavior, particularly at later developmental stages. For instance, loss of *oxtr or oxtrl* increases inter‐individual distance and reduces shoal cohesion and coordinated swimming at 8 weeks post‐fertilization (wpf), but not at 4 wpf, indicating specific roles in the maturation of group behavior. Additionally, Ribeiro, Nunes, Teles, et al. (2020) showed that deficits in social recognition in *oxtr* mutants persist regardless of the social environment, while other behaviors such as social habituation and group integration are modulated by the genotypic composition of the shoal, highlighting the functional and behavioral relevance of the receptors at both individual and group levels.

In zebrafish, OXTRs are widely expressed in brain areas involved in sensory processing and in structures that comprise the social decision‐making network (SDMN), such as: Dm (medial pallium), Dl (lateral pallium), Vd (dorsal nucleus of subpallium), Vc (central part of subpallium), TPp (periventricular nucleus of the posterior tuberculum), Vv (ventral part of subpallium), Vl (lateral subpallium), Vs (supracommissural nucleus of subpallium), POA (preoptic area), PAG (periaqueductal gray substance), VnT (ventral tuberal nucleus), and aNT (anterior tuberal nucleus) (O'Connell and Hofmann 2012; Grinevich et al. 2016; Johnson and Young 2017; Geng and Peterson 2019). OXTRs expressed in these brain structures form interconnected circuits that can modulate a range of social adaptive behaviors, such as affiliative behaviors, social salience, motivation, and reward (Grinevich et al. 2016; Geng and Peterson 2019). Along these lines, an interesting study in zebrafish showed that phenotypic components of social behavior, including motivation and anxiety, are associated with genetic polymorphism in *oxtr* genes (Kareklas, Teles, Nunes et al. 2023).

**FIGURE 1:**
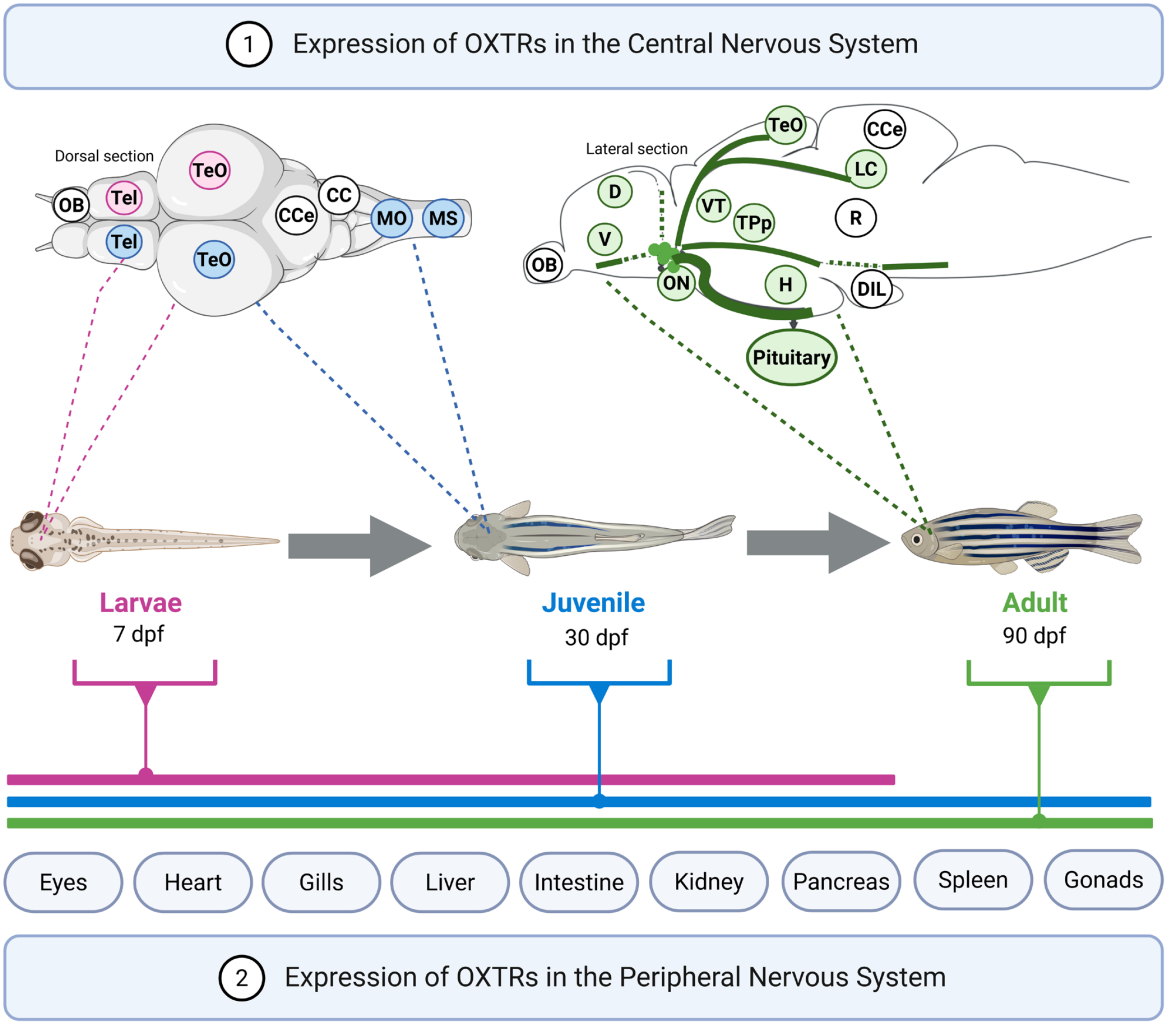
Schematic representation of oxytocin receptors expression throughout the zebrafish development. (1) Oxytocin receptors (OXTRs) expression in the central nervous system. At the larval stage (7 dpf), OXTRs are expressed in the telencephalon (Tel) and optic tectum (TeO). At the juvenile stage (30 dpf), OXTRs are expressed in Tel, TeO, medulla oblongata (MO) and medial septum (MS). In the adult stage (90 dpf), oxytocin projects to brain areas including the dorsal telencephalic area (D), hypothalamus (H), ventral telencephalic area (V), olfactory bulb (OB), optic nerve (ON), TeO, periventricular nucleus of the posterior tubercle (TPp), ventral thalamus (VT), locus coeruleus (LC) and pituitary. (2) OXTRs expression in the peripheral nervous system. In the larval stage, OXTRs are found in eyes, heart, gills, liver, intestine, kidneys, and pancreas. In juvenile and adult fish, OXTRs expression is also observed in the aforementioned organs, as well as in the spleen and gonads. CC = crista cerebralis, CCe = cerebellar body, D = dorsal telencephalic area, DIL = diffuse nucleus of the inferior lobe, dpf = days post fertilization, H = hypothalamus, LC = locus coeruleus, MO = medulla oblongata, MS = spinal cord, OB = olfactory bulb, ON = optic nerve, *R* = raphe nucleus, Tel = telencephalon, TeO = optic tectum, TPp = periventricular nucleus of the posterior tubercle, V = ventral telencephalic area, VT = ventral thalamus, Pituitary. Figure created with BioRender.

In the zebrafish brain, oxytocin neurons have complex projections from early developmental stages (Figure 1). Projections that innervate the pituitary begin on the third dpf, whereas those targeting other regions such as optic tectum, hypothalamus, and ventral telencephalon emerge between four and six dpf and remain stable until eight dpf (Herget et al. 2017). It has been reported that oxytocinergic neurons project to the hindbrain and spinal cord after 5 dpf, and between 6 and 8 dpf, oxytocin modulates nocifensive behavior through premotor targets in the brainstem (Wircer et al. 2017; Wee et al. 2019).

In adult zebrafish, oxytocin neurons make projections in several regions, including the ventral nucleus of the ventral telencephalon (Vv), the previous part and posterior part of the parvocellular preoptic nucleus (PPa and PPp), periventricular nucleus of the posterior tubercle (TPp), and preglomerular nuclei (PGm). Oxytocin signaling can regulate the anxiety and aggression levels in those regions by different environmental stressors in the adult zebrafish (Chuang et al. 2021). It has also been demonstrated that early disturbance (4.5 and 6 dpf) in oxytocin neurons has structural effects in the adult stage, which can be irreversible, including deficits in social affiliation, damage in the development of subsets of dopaminergic neurons, and changes in brain activity in the Vv and PPa nuclei. These changes can lead to a decreased response to social stimuli and changes in connectivity patterns in the SDMN (Nunes et al. 2021).

## 3 The Role of Oxytocin Signaling in ASD: Insights From Zebrafish Models

Sociability, a complex trait which involves both motivational and cognitive processes, is a fundamental component of social behavior in humans and animals (Rappeneau and Castillo Díaz 2024). Some key aspects of sociability, such as preference for social interaction, group cohesion, and the ability to recognize and respond to conspecifics, are social behaviors evolutionary shared across species, including zebrafish (Gerlai 2014; Ribeiro, Nunes, Gliksberg, et al. 2020; Usui 2024). In both humans and zebrafish, living in groups can provide adaptive advantages, improving foraging strategies, reproductive success, predator avoidance, and social learning. Furthermore, social interaction facilitates the establishment of affective bonds, which are crucial for individual well‐being and group stability (Harpaz and Schneidman 2020; Lucore and Connaughton 2021; Pérez‐Manrique and Gomila 2021).

In humans, social interactions are highly complex, integrating language, emotion, anxiety, cognition, and cultural norms to facilitate nuanced communication and relationship‐building. In contrast, in animal species such as zebrafish, social behavior is primarily driven by shoaling and schooling, which are based on visual cues and rapid, coordinated movements that promote survival (Stewart, Braubach, et al. 2014; Usui 2024). Despite differences in complexity, zebrafish exhibit key social behaviors that are analogous to human sociability. For instance, zebrafish are social animals that prefer group living and are able to discriminate between familiar and unfamiliar conspecifics, suggesting a capacity for social recognition (Oliveira 2013; Abril‐de‐Abreu et al. 2015; Hinz and de Polavieja 2017; Silva et al. 2019). Furthermore, when zebrafish are under conditions of fear and stress, they can react equally to distress and suffering exhibited by their conspecifics (Oliveira et al. 2017; Burbano Lombana et al. 2021).

Evidence suggests that both humans and zebrafish share similar neural circuits within the oxytocinergic pathway, which are involved in regulating social behavior (Knobloch and Grinevich 2014; Grinevich et al. 2016; Matsushita and Nishiki 2025). Supporting this, studies in zebrafish demonstrated that their orthologs of oxytocin (isotocin) and vasopressin participate in regulating social preference, group cohesion, and reproductive behavior (Braida et al. 2012; Altmieme et al. 2019). In addition, disruptions in the oxytocin system have been linked with social disorders such as ASD (Hasan 2024; Matsushita and Nishiki 2025).

ASD is a disorder which comprises neurodevelopmental disorders (NDDs), a group of conditions that disrupt critical periods of brain development, usually emerging during early childhood and characterized by impairments in cognitive, emotional, and motor development (Parenti et al. 2020). ASD, one of the most prevalent NDDs, is defined by two core symptom domains, which include persistent deficits in social interaction and communication, and restricted, repetitive patterns of behavior. These symptoms negatively impact daily functioning, reducing the quality of life for individuals with ASD (Sánchez Amate and de la Luque Rosa 2024; Jain et al. 2025). Furthermore, social deficits in ASD are associated with dysregulation of the oxytocin system during early neurodevelopment, which may lead to altered synaptic plasticity in key brain regions involved in the modulation of social behavior, including the amygdala, prefrontal cortex, and ventral striatum (Rajamani et al. 2018).

One of the most well‐established zebrafish models of ASD involves exposure to sodium valproate (VPA), a drug used to treat mood disorders and epilepsy, which is known to induce ASD‐like phenotypes (Flores‐Prieto et al. 2024; Camussi et al. 2025). In zebrafish, several behavioral phenotypes are related to core symptoms of ASD, such as deficits in social interaction or shoaling, and repetitive behaviors. Moreover, other behavioral phenotypes mimic ASD‐associated comorbidities, including anxiety‐like behavior, aggression, and impairments in learning and memory (Stewart, Braubach, et al. 2014; Stewart, Nguyen, et al. 2014; Meshalkina et al. 2018; Pal et al. 2025). Specifically, social interaction and shoaling are assessed by measuring the preference of zebrafish to interact with conspecifics, while repetitive behaviors are evaluated by quantifying the frequency of stereotypic swimming patterns (e.g., circling) in the open tank task (Stewart, Nguyen, et al. 2014; Pal et al. 2025). Regarding comorbidities, the novel tank test (NTT) and light–dark test are widely used to assess anxiety‐like states. In addition, the mirror test and the avoidance inhibitory task (or novel object recognition) are established methods for evaluating aggression and cognitive deficits (Stewart, Braubach, et al. 2014; Pal et al. 2025).

In this context, studies have observed that VPA exposure during the embryonic period (0 to 48‐ or 120‐h post fertilization) does not affect the survival and morphological development of zebrafish larvae (Zimmermann et al. 2015; Lee et al. 2018). Embryos treated with VPA for 48 hpf exhibited social deficits that were attenuated by oxytocin (25, 50, or 100 μM) administered for 24 or 48 h. Oxytocin, at 50 μM for 48 h, was most effective, increasing conspecific contact, reducing anxiety‐like behavior, and enhancing social interaction (Rahmati‐Holasoo et al. 2023). Oxytocin also reversed VPA‐induced downregulation of *shank3a, shank3b*, and *oxtr* gene expression, suggesting its potential to modulate synaptic functions and oxytocinergic signaling in ASD‐related zebrafish phenotypes. On the other hand, embryos exposed to VPA at a concentration of 100 μM show reduced hatching rates (Lee et al. 2018). Moreover, the embryonic VPA exposure induces ASD‐related behavior phenotypes, including hyperlocomotion at 6 dpf and increased anxiety‐like behavior at 6, 70, and 120 dpf (Zimmermann et al. 2015).

In this way, embryonic VPA exposure induces persistent deficits in social interaction that are evident at both 70 and 120 dpf (Zimmermann et al. 2015). Interestingly, despite these social impairments, VPA‐treated zebrafish did not exhibit significant differences in aggressive behavior in the mirror test when compared to control animals at the same developmental stages (Zimmermann et al. 2015). The behavioral findings mentioned above are supported by molecular evidence, demonstrating that morpholino‐mediated knockdown of *shank3*, one of the most clinically relevant genes associated with the genetic etiology of ASD, recapitulates core ASD‐like phenotypes in zebrafish, including reduced social interaction and increased repetitive swimming behaviors (Liu et al. 2018). Furthermore, VPA exposure in the embryonic stage induces transcriptional alterations in multiple ASD‐related genes such as *shank3a, shank3b*, *adsl*, *mbd5a*, *tsb1b*, and *oxtr* (Lee et al. 2018; Rahmati‐Holasoo et al. 2023). Taken together, these findings suggest an impairment in ASD‐associated genetic pathways and the validity of the VPA exposure model to mimic core behavioral and molecular hallmarks of ASD pathophysiology.

Similar to humans, the oxytocinergic system in zebrafish also appears to modulate ASD‐related social behaviors. A recent study investigated the effects of different oxytocin exposure protocols—continuous exposure (Con_OT group), 15‐min daily exposure (15M_OT group), and 15‐min exposure every 2 days (2D_OT group)—on behavioral parameters in adult zebrafish over a 7‐day period (Robea et al. 2024). The authors observed that the 15M_OT group showed increased locomotor activity during a 3‐day period. In addition, the 15M_OT and 2D_OT groups demonstrated reduced aggression during the first 3 days of treatment. These findings suggest that the behavioral effects of oxytocin are time and exposure‐dependent, reinforcing its potential as a modulator of ASD‐related social phenotypes (Robea et al. 2024).

A study using *oxtr* and *oxtrl* knockout zebrafish investigated the role of oxytocin receptors in the development of social behavior. While wild‐type (WT) zebrafish exhibited a gradual increase in social preference from 2 to 4 weeks post‐fertilization (wpf), remaining stable up to 8 wpf, knockout fish exhibited an earlier peak at 3 wpf, followed by a decline (Gemmer et al. 2022). Additionally, both *oxtr* and *oxtrl* mutants demonstrated increased susceptibility to isolation‐induced social deficits at 8 wpf (Gemmer et al. 2022). These findings suggest that the oxytocin receptors Oxtr and Oxtrl are essential not only for the proper early development of social behavior but also for its maintenance over time. The absence of these receptors leads to an early emergence of social behavior but compromises its long‐term stability, particularly under conditions of social isolation (Gemmer et al. 2022).

Corroborating previous studies, the pharmacological blockade of the OXTR using the selective antagonist L‐368899 significantly reduced social preference in both larval and adult zebrafish. However, this antagonist did not alter the anxiety‐like behavior in adult zebrafish (Landin et al. 2020). Another study investigated the interaction between glutamatergic and oxytocinergic systems in ASD phenotypes induced by MK801, a non‐competitive NMDA receptor antagonist. MK‐801 exposure decreases social interaction and aggression, and these deficits were reversed by oxytocin administration (Zimmermann et al. 2016). Carbetocin, an oxytocin receptor agonist, restored normal social and aggressive behaviors, whereas the antagonist L‐368899 failed to reverse MK‐801‐induced deficits (Zimmermann et al. 2016).

Oxytocin signaling in zebrafish larvae modulates not only social interaction but also social cognition and adaptive behavior. Specifically, oxytocin integrates social cues with nociceptive and appetite‐driven behaviors to enhance survival (Wee et al. 2019, 2022). It also contributes to recognition memory, enabling the discrimination between familiar and novel stimuli, as well as visual processing of movement and shape during social interactions (Nunes et al. 2020; Ribeiro, Nunes, Gliksberg, et al. 2020). These studies emphasize oxytocin as a broad neuromodulator, underscoring its central role in neurodevelopment.

These findings show that zebrafish is a robust model for investigating behavioral, neurobiological, and molecular mechanisms underlying ASD. However, despite the utility of zebrafish in studying social behavior and oxytocinergic signaling, surprisingly few studies have explored the role of the oxytocinergic system, as well as drugs oxytocinergic modulators in ASD using this animal model. This gap underscores a need for further research focused on oxytocinergic signaling on ASD‐like phenotypes in zebrafish, which may contribute to therapeutic advances for NDDs.

## 4 Evaluating Translational Relevance: Strengths and Limitations of Zebrafish Behavioral Assays for ASD Research

Over the past decades, the clinical understanding and classification of ASD have evolved significantly, with current definitions in the DSM‐5 and ICD‐11 (Ousley and Cermak 2014) recognizing ASD as a heterogeneous condition with a broad range of behavioral manifestations (Tidmarsh and Volkmar 2003; World Health Organization 2019). While these revisions have improved diagnostic precision, they have also complicated comparisons across studies and time, underscoring the need for integrative and standardized approaches in ASD research. Although zebrafish lack the complex cognitive functions and verbal communication found in humans, several behavioral domains relevant to ASD can be reliably assessed using validated paradigms (Table 1). However, some limitations need to be discussed, particularly regarding the sensitivity of each behavioral assay in the zebrafish model and its specificity to ASD‐related phenotypes.

**TABLE 1:** Behavioral Tests in Zebrafish for Investigating ASD phenotypes.

Behavioral test	Developmental stage	Description	Altered endpoints in ASD models	References
Optomotor response (OMR)	Larvae (from ~6 dpf) and juveniles	Assesses larval reflexive responses to moving visual stimuli through two tasks: non‐aversive (visuomotor integration/tracking) and aversive (escape/threat avoidance). Distinguishes innate tracking from defensive behaviors.	In models with dysfunction (genetic or pharmacological exposure), a reduction/alteration in stimulus‐following rate and increased latency is observed. Visual or sensorimotor integration deficits. Embryonic VPA exposure (25 μM): VPA led to changes in retinal development, OMR deficits, and alterations in sleep, indicating that part of the ASD‐like phenotype may reflect sensory/visual deficits in addition to social alterations.	Basnet et al. 2019; Crowley‐Perry et al. 2021; Harpaz et al. 2021; Nabinger et al. 2021; De Oliveira‐Mello et al. 2023
Novel tank test (NTT)	Larvae, juveniles, and adults	Evaluates exploratory and anxiety‐like behavior through multiple endpoints: locomotion (distance, velocity, erratic movements), bottom‐dwelling (anxiety‐like behavior), and freezing (stress‐related responses).	Larvae—VPA (48 μM): Increased distance traveled and entries in the outer zone. Adults—VPA (48 μM): No locomotor changes, but increased time in the bottom zone (anxiety‐like behavior). Adults—Shank3 models: Increased time in the bottom zone and altered locomotor activity.	Liu et al. 2018; Liu et al. 2021; Zimmermann et al. 2015
Social interaction	Larvae, juveniles, and adults	Measures innate sociability based on preference to interact with a conspecific shoal (live or video). Key metrics: distance to shoal and interaction time. Reduced proximity indicates social deficits.	Adult—Shank3/Shank3b: Exhibited social deficits. Adult—VPA (48 μM): Reduced interaction time. Larval—VPA (48 μM): 50 and 100 μM oxytocin (24‐48 h) increased social interaction in VPA models. Adult—VPA (48 μM): Increase in social interaction time and reversal of behavioral deficits induced by treatments that mimic aspects of ASD. Adult—DYRK1A KO: Spent less time in the social approach zone. Juvenile—Poly(I:C)/MIA: Reduced sociability	Liu et al. 2018; Liu et al. 2021; Zimmermann et al. 2015; Rahmati‐Holasoo et al. 2023; Kim et al. 2017; Wu et al. 2023
Shoaling	Larvae and adults	Assesses sociability and group cohesion (schooling). Measures average inter‐individual distance, group area, and proximity to conspecifics.	Larvae—VPA (48 μM): Oxytocin treatment (50 e 100 μM by 24 and 48 h exposure) increased social interaction. Larva e Juvenile—VPA (48 μM): Oxytocin (50 μM) increased the frequency and time of contact between conspecifics. Adult—VPA (48 μM): Reduced time near conspecifics and fewer social approaches. Adult—Shank3‐deficient models: Exhibited reduced cohesion.	Rahmati‐Holasoo et al. 2023; Camussi et al. 2025; Liu et al. 2021; Chen et al. 2018
Inhibitory avoidance task	Adults	Assesses emotional learning and memory using a visual cue paired with an electric shock. Measures escape latency, freezing, and immobility. Effective for assessing long‐term memory/threat association.	Gap: Currently, there is a gap in the literature regarding studies combining ASD models (genetic/pharmacological) with this specific test.	Blank et al. 2009; Meshalkina et al. 2018
Object recognition memory	Larvae and adults	Evaluates discrimination between familiar and novel objects (or conspecifics) after habituation. Preference for novelty indicates intact memory.	Adult—OXTR−/− (Oxytocin receptor mutant): Impaired novel object recognition, linking OXTR to recognition memory. *Larvae—kcc2a* KO zebrafish: Spent less time exploring the novel object compared to the control group.	Ribeiro, Nunes, Gliksberg, et al. 2020; Ribeiro, Nunes, Teles, et al. 2020; Naderi et al. 2024
Social contagion of fear	ts Adults	Evaluates emotional contagion by exposing fish to conspecifics (real or video) under stress (alarm substance). Measures swimming speed, erratic movements, and freezing. Reveals lack of emotional synchronization and failures in affective contagion/empathy in ASD models.	Shank3a mutation: Reduction in social fear contagion (less “imitation” of distress).	Kareklas, Teles, Dreosti, and Oliveira 2023; Kareklas, Teles, Nunes, and Oliveira 2023; Oliveira et al. 2017; Silva et al. 2019; Burbano Lombana et al. 2021; Akinrinade, Kareklas, et al. 2023; Akinrinade, Varela, et al. 2023
Aggressive behavior	Larvae, juveniles, and adults	Assessed through mirror tests or conspecific encounters. Parameters: biting, chasing, lateral displays, and circling. This task highlights difficulties in social hierarchy modulation.	Larvae—VPA (48 μM): Increased number of attacks/min. Adult—VPA: Typical agonistic behaviors (confrontations/territorial disputes) Larvae—Oxytocin: Increased attack frequency in VPA models.	Zimmermann et al. 2015; Rahmati‐Holasoo et al. 2023; Zabegalov et al. 2019
Light–dark test	Larvae	Assesses anxiety and exploration (scototaxis). Measures time spent in light vs. dark, entries, and latency.	Larvae—VPA (48 μM): Altered crossing times and % time in light. Larvae—VPA (50 μM): Increased total distance in dark compartment Larvae—Oxytocin: Increased crossings between compartments.	Lee et al. 2018; Rahmati‐Holasoo et al. 2023; Golla et al. 2020; Zhan, Li, et al. 2024
Repetitive behavior	Adults	Analyzes stereotypic patterns (e.g., circling) using novel tank paradigms with detailed tracking.	Adult—Shank3 deficiency: Repetitive circular swimming. Gut‐Brain Axis: Circular swimming and stereotypic behaviors observed in models studying enteric nervous system/immune interactions.	Liu et al. 2021; Tayanloo‐Beik et al. 2022; Andersen‐Civil et al. 2023

The behavioral repertoire described for zebrafish can be applied to model and evaluate translational behaviors relevant to ASD. As mentioned above, the optomotor response, which is usually used to evaluate the sensorimotor integration and visual‐motor function, aligns with sensory processing deficits observed in individuals with ASD (Patten et al. 2013). Despite its important sensorimotor effects, this test does not assess affective states; therefore, additional tests are needed. Although the subjective dimension of emotion cannot be directly assessed in zebrafish, well‐validated behavioral paradigms allow for the inference of emotional/affective states through measurable outputs. Key assays include the inhibitory avoidance task, light/dark preference test, fear contagion paradigm, exposure to predator cues, cognitive bias assays, and the mirror test (von Trotha et al. 2014; De Abreu et al. 2020; Akinrinade, Varela, et al. 2023; Gazzano et al. 2025; Zhdanov et al. 2025). Collectively, these tools evaluate phenotypes related to anxiety, fear, stress, aversion, cognitive bias, and aggression, providing insights into the emotional/affective‐like behavior in zebrafish. Moreover, oxytocin administration in zebrafish may lead to atypical responses in this test. While the test itself does not directly assess emotional behavior, the administration of oxytocin could influence the fish's decision‐making or behavior, thereby affecting the test outcome, even in the absence of emotional involvement, reinforcing the importance of this neurohormone in modulating ASD‐like behavior.

A similar issue arises when considering the NTT; however, rather than viewing individual variability as a confounding factor masked by group averages, the NTT should be recognized for its ability to identify distinct individual phenotypes. This is a crucial consideration given the heterogeneity of ASD. Furthermore, tracking individual data points across the NTT and concurrent behavioral assays allows for a more nuanced analysis that captures the spectrum nature of the condition. Upon exposure to a novel tank, fish typically show anxiety‐like behaviors such as geotaxis (bottom‐dwelling), which progressively diminishes as they begin to explore the environment (Mocelin et al. 2015). The exploratory response is modulated by anxiety, fear, and pharmacological manipulation targeting systems such as the oxytocin pathway. Although studies utilizing the direct waterborne administration of the neuropeptide (Isotocin) are limited, the oxytocinergic signaling pathway is well‐established as a modulator of anxiety and social behavior in this model. Recent research conducted by Maciag et al. (2025) demonstrated that pharmacological activation of oxytocin receptors in zebrafish using the agonist WAY‐267464 resulted in a reduction in thigmotactic behavior (an anxiety indicator in the Novel Tank Test) and an increase in the social preference index. These results confirm that modulation of this pathway has anxiolytic and prosocial effects, providing mechanistic support for the role of oxytocin signaling in behavioral assays (Maciag et al. 2025). NTT could be well‐suited to identify individual differences, particularly when used in combination with other behavioral assays. Tracking individual data points across tests could strengthen interpretation and allow for a more nuanced analysis aligned with the heterogeneity of ASD.

To complement the optomotor response and novel tank test, social interaction assays are particularly relevant for modeling ASD in zebrafish and can be used to understand the group cohesion responses (Gerlai 2014). These behaviors depend on intact locomotor function to avoid false positive/negative behavior. In addition, it is not always possible to separate visual attraction from true social motivation. Oxytocin has been shown to reestablish social interaction behavior in zebrafish, reinforcing its potential role in treating ASD‐related social impairments (Landin et al. 2020).

An additional test that may be useful for assessing ASD‐like behavior is the inhibitory avoidance test, which introduces an emotionally salient stimulus. This test evaluates aversive learning and memory by conditioning the animal to avoid a specific area through the application of an electric shock (Tayanloo‐Beik et al. 2022). It may be particularly suitable for investigating the influence of oxytocin on memory and learning, especially in aversive contexts, as it involves the animal's learned avoidance of a location due to the ‘emotional risk’ of receiving another shock. In contrast, the Novel Object Recognition (NOR) test evaluates object recognition by training zebrafish to explore familiar versus novel objects (Gaspary et al. 2018). Unlike the Inhibitory Avoidance test, NOR does not involve aversive stimuli and can be adapted by presenting objects with distinct colors and shapes and can be adapted to explore perceptual and cognitive processes relevant to ASD (Gaspary et al. 2018). Interestingly, zebrafish mutants lacking a functional oxytocin receptor—despite showing a preference for social interaction in shoals—do not exhibit a preference for familiar conspecifics, suggesting that oxytocin is critical for social recognition and reinforcing its essential role in promoting appropriate social behavior (Ribeiro, Nunes, Gliksberg, et al. 2020).

Studies highlight oxytocin's broader impact on social cognition (Akinrinade, Kareklas, et al. 2023; Kareklas, Teles, Dreosti, et al. 2023). These findings aligned with recent evidence suggest that oxytocin is not only involved in social preference but also in more complex forms of social cognition, such as emotional contagion (Akinrinade, Kareklas, et al. 2023). For instance, emotionally distressed demonstrator zebrafish can elicit fear‐related behaviors in observer fish, a phenomenon known as social fear contagion. Importantly, this response is abolished in oxytocin receptor mutants, indicating that this neurohormone is necessary for the transmission of affective states between conspecifics (Akinrinade, Kareklas, et al. 2023). These observations reinforce the idea that oxytocin is a key modulator of basic empathic processes in vertebrates (Akinrinade, Kareklas, et al. 2023).

In this context, aggression emerges as a behavioral domain potentially influenced by deficits in social cognition and emotional regulation, two features frequently associated with empathy impairments. Although aggression is not a core diagnostic criterion of ASD, increased irritability, impulsivity, and reactive aggression are commonly reported in a subset of individuals, particularly in response to frustration, sensory overload, or social misunderstanding. These manifestations are thought to reflect, at least in part, impaired emotional self‐regulation and social processing (Fitzpatrick et al. 2016).

When considering the zebrafish as a translational model, aggression is typically assessed using paradigms such as the mirror‐induced aggression test, where individuals display threat or attack behaviors towards their own reflection (Oliveira et al. 2011). However, quantifying aggression in zebrafish presents several challenges and interpretation is limited by the species' lack of cognitive intentionality and whether aggressive responses reflect territoriality, fear, social dominance, or stress (Freudenberg et al. 2016). Furthermore, aggression in zebrafish is context‐dependent, varying with factors such as prior social experience, tank environment, and genetic background (Zabegalov et al. 2019). Nonetheless, modulation of aggression by oxytocin or its analogs may provide additional information about the neurochemical pathways related to social dysregulation (Zimmermann et al. 2016; Robea et al. 2024). The translational perspective is relevant, as human studies indicate that oxytocin administration intensifies aggressive responses specifically following provocation or social rejection (Pfundmair et al. 2018), suggesting that the neuropeptide acts as an amplifier of social salience in threatening contexts. Furthermore, this pro‐aggressive effect of oxytocin was observed exclusively in participants with low trait anxiety, suggesting that the neurochemical pathways underlying social dysfunction involve a complex interaction between the oxytocinergic system, negative social stimuli, and individual baseline disposition (Pfundmair et al. 2018). In summary, while zebrafish may not recapitulate the full complexity of aggression observed in ASD, their utility lies in dissecting conserved molecular and neurochemical pathways, such as those mediated by oxytocin, that underlie social and emotional regulation.

## 5 Advantages, Limitations, and Ethical Challenges of Using Zebrafish in ASD

Zebrafish continue to emerge as a powerful model organism for ASD research due to several advantages, including high genetic and neurochemical similarity to humans, high fecundity, and embryonic transparency. The latter permits the manipulation and direct visualization of the central nervous system, thereby facilitating the study of neurodevelopmental disorders. Additional advantages, such as rapid drug absorption and a well‐characterized behavioral repertoire that recapitulates core ASD phenotypes, enable high‐throughput pharmacological screening (Stewart, Braubach, et al. 2014; Stewart, Nguyen, et al. 2014; Fontana et al. 2019; Gerlai 2023). Further advantages concerning therapeutic interventions targeting the oxytocinergic system are detailed in Table 2.

**TABLE 2:** Advantages of the zebrafish model to study the oxytocinergic system in ASD.

Advantages	Description/Evidence	References
Identification of therapeutic targets	Oxytocin receptors (Oxtr, Oxtrl) modulate social behaviors in zebrafish; genetic or pharmacological manipulation has been shown to reverse social deficits, validating these targets.	Landin et al. 2020; Gemmer et al. 2022; Rahmati‐Holasoo et al. 2023; Zhang, Mi, et al. 2024; Robea et al. 2024
Development of synthetic agonists	Non‐peptide oxytocin agonists (e.g., LIT‐001) and analogues enhance social interaction. The model allows for rapid screening to explore molecular mechanisms and dose–response proflies.	Tayanloo‐Beik et al. 2022; Robea et al. 2024; Zhang, Li, et al. 2024
Personalized administration strategies	Timing and duration of administration influence outcomes; early interventions during critical developmental periods produce longer‐lasting effects.	Ribeiro, Nunes, Gliksberg, et al. 2020; Ribeiro, Nunes, Teles, et al. 2020; Rahmati‐Holasoo et al. 2023
General translational utility	High genetic and neurochemical conservation (~70% shared genes) combined with transparent embryos allows direct observation of neural development. Quantifiable social behaviors facilitate rapid drug screening and the assessment of early interventions.	Gemmer et al. 2022; Tayanloo‐Beik et al. 2022
Bridging translational gaps	While direct translation to humans requires caution due to interspecies differences, zebrafish studies provide relevant information into oxytocinergic pathways and pathophysiological mechanisms, serving as a bridge between in vitro and mammalian studies.	Ribeiro, Nunes, Gliksberg, et al. 2020; Ribeiro, Nunes, Teles, et al. 2020; Tayanloo‐Beik et al. 2022; Rahmati‐Holasoo et al. 2023; Robea et al. 2024; Zhang, Mi, et al. 2024

However, despite the aforementioned strengths of the zebrafish ASD model, it is crucial to address some concerns through a critical analysis of data interpretation and translational potential (bench to bedside). The main limitations include genome duplication, differences in the blood–brain barrier (which can affect drug permeability), the late development of social behavior (around 14 days post‐fertilization), the absence of parental care, and challenges regarding reproducibility. Furthermore, variability in environmental conditions and husbandry practices, the lack of subjective responses in behavioral tests, and the absence of vocal communication must be considered (Stewart, Braubach, et al. 2014; Stewart, Nguyen, et al. 2014; Gerlai 2023). These limitations open key critical questions (Table 3).

**TABLE 3:** Open questions in the ASD zebrafish model.

How does teleost genome duplication affect the functional redundancy and pleiotropy of ASD‐risk gene orthologs?
How do different genetic ASD backgrounds influence the severity of behavioral phenotypes in ASD models?
What epigenetic markers are altered in transgenerational zebrafish ASD genetic and pharmacological models?
How can we assess robust behavioral biomarkers to evaluate the affective/emotional response in zebrafish ASD models?
How do environmental factors (e.g., drug exposure, parental effects, gestational nutrition, pesticides) interact with genetic factors to modulate the severity of core and comorbid ASD phenotypes?
How do strain, husbandry conditions, and sex differences impact the reproducibility and replicability of ASD phenotypes?
How do strain, husbandry conditions, and sex differences influence the efficacy of pharmacological interventions on ASD phenotypes?

Finally, ethical challenges inherent to modeling ASD in zebrafish also need to be addressed. The main concerns fall into the following categories: (i) animal welfare, including minimizing distress and pain, and adhering to the principles of the 3Rs (Replacement, Reduction, and Refinement); (ii) scientific principles, such as the appropriate choice of developmental stage (embryo versus adult), study pre‐registration, open access data reporting, and ensuring robustness and reproducibility; and (iii) conduct principles, promoting a culture of care and ethical stewardship towards the animals used in research.

## 6 Conclusions and Future Directions

Although there is a wide scope of neurochemical and behavioral factors implicated in NDDs, there is an imminent need for further research in this field. Current therapeutic treatments for these disorders may not effectively address the core symptoms due to the focus on treating comorbidities, as observed in ASD. In this context, zebrafish have emerged as a powerful and versatile translational model. Their high genetic and physiological homology with mammals, transparency during early development, ease of genetic manipulation, and suitability for high‐throughput screening make them a compelling system for probing the neurobiological basis of NDDs (Washbourne 2023). While zebrafish cannot fully replicate the complexity of human behavior, their conserved neural circuits and robust social repertoire enable the study of evolutionarily preserved mechanisms, particularly those modulated by neuropeptides such as oxytocin.

Findings from zebrafish models suggest that oxytocin signaling influences not only social preference and recognition but also higher‐order behaviors such as social fear contagion and aggression regulation. These results, parallel to the findings in rodent and human studies, highlight the translational potential of oxytocin‐targeted interventions. Future research should focus on several ways:

- Elucidating oxytocinergic circuitry in zebrafish through imaging and optogenetic tools, to map functional connectivity relevant to social and emotional processing.
- Developing and validating behavioral paradigms that analyze ASD‐like phenotypes in zebrafish, including individual‐based assessments that show behavioral heterogeneity.
- Integrating multi‐omics approaches (e.g., transcriptomics, proteomics, epigenomics) to identify oxytocin‐regulated molecular networks and their dysregulation in NDDs.
- Evaluating gene–environment interactions, particularly how early‐life stressors or social deprivation may modulate oxytocin signaling and their behavioral consequences.
- Advancing drug discovery using zebrafish‐based platforms to screen oxytocinergic modulators and test their efficacy in behavioral domains, with potential for translation into mammalian models and clinical trials.

In summary, zebrafish provide a unique platform to deepen our understanding of the neurobiological substrates of NDDs. Future research in this field may contribute to the development of more effective treatments that address the core deficits of NDDs and improve the quality of life for affected individuals.

## Author Contributions

**Géssica Peres:** conceptualization, investigation, writing – original draft, methodology, writing – review and editing. **Melissa Talita Wiprich:** writing – review and editing, investigation. **Darlan Gusso:** investigation, writing – review and editing. **Carla Denise Bonan:** conceptualization, writing – review and editing, supervision.

## Funding

This work was supported by Conselho Nacional de Desenvolvimento Científico e Tecnológico, 306115/2023‐9, 402097/2023‐8. Coordenação de Aperfeiçoamento de Pessoal de Nível Superior, 001, 88887.883378/2023‐00.

## Conflicts of Interest

The authors declare no conflicts of interest.

## Data Availability

The authors have nothing to report.
